# MicroRNA-203 impacts on the growth, aggressiveness and prognosis of hepatocellular carcinoma by targeting *MAT2A* and *MAT2B* genes

**DOI:** 10.18632/oncotarget.26838

**Published:** 2019-04-19

**Authors:** Maria M. Simile, Graziella Peitta, Maria L. Tomasi, Stefania Brozzetti, Claudio F. Feo, Alberto Porcu, Antonio Cigliano, Diego F. Calvisi, Francesco Feo, Rosa M. Pascale

**Affiliations:** ^1^ Department of Medical, Surgical and Experimental Sciences, Division of Experimental Pathology and Oncology, University of Sassari, Sassari, Italy; ^2^ Department of Medicine, Cedars-Sinai Medical Center, Los Angeles, CA, USA; ^3^ Department of Surgery “Pietro Valdoni”, University of Rome “La Sapienza”, Rome, Italy; ^4^ Department of Medical, Surgical and Experimental Sciences, Division of Surgery, University of Sassari, Sassari, Italy; ^5^ Institute of Pathology, University of Regensburg, Regensburg, Germany

**Keywords:** methionine metabolism, methyladenosyltransferases, S-adenosylmethionine, HCC prognosis, HCC therapy

## Abstract

Hepatocellular carcinoma (HCC) is characterized by the down-regulation of the liver-specific methyladenosyltransferase 1A (*MAT1A*) gene, encoding the S-adenosylmethionine synthesizing isozymes MATI/III, and the up-regulation of the widely expressed methyladenosyltransferase 2A (*MAT2A*), encoding MATII isozyme, and methyladenosyltransferase 2B (*MAT2B*), encoding a β-subunit without catalytic action that regulates MATII enzymatic activity. Different observations showed hepatocarcinogenesis inhibition by miR-203. We found that miR-203 expression in HCCs is inversely correlated with HCC proliferation and aggressiveness markers, and with *MAT2A* and *MAT2B* levels. MiR-203 transfection in HepG2 and Huh7 liver cancer cells targeted the 3’-UTR of *MAT2A* and *MAT2B*, inhibiting *MAT2A* and *MAT2B* mRNA levels and MATα2 and MATβ2 protein expression. These molecular events were paralleled by an increase in SAM content and were associated with growth restraint and apoptosis, inhibition of cell migration and invasiveness, and suppression of the expression of *CD133* and *LIN28B* stemness markers. In contrast, *MAT2B* transfection in the same cell lines led to a rise of both MATβ2 and MATα2 expression, associated with increases in cell growth, migration, invasion and overexpression of stemness markers and p-AKT. Altogether, our results indicate that the miR-203 oncosuppressor activity may at least partially depend on its inhibition of *MAT2A* and *MAT2B* and show, for the first time, an oncogenic activity of *MAT2B* linked to AKT activation.

## INTRODUCTION

Hepatocellular carcinoma (HCC) is a frequent and fatal human cancer, with 0.25–1 million newly diagnosed cases each year [1]. Complex relationships among genetic, etiologic, and environmental risk factors determine a genotypic and phenotypic heterogeneity within human HCC [1]. Previous observations showed a decrease of the major methyl donor SAM (S-adenosylmethionine) along hepatocarcinogenesis, and the preventive effects of the reconstitution of SAM pool [2]. SAM is synthesized by methyl-adenosyltransferases. Liver-specific *MAT1A* (methyladenosyltransferase 1A) encodes the isozymes MATI/III, tetramer and dimer of the α1 subunit, respectively [3]. *MAT2A* (methyladenosyltransferase 2A) encodes α2 subunit, the widely distributed enzyme MATII isoform. *MAT2A* expression prevails in fetal liver and is substituted by *MAT1A* in adult liver [3]. A third gene, *MAT2B* (methyladenosyltransferase 2B), encodes a β-subunit without catalytic action, which regulates MATII by lowering its Km for methionine and Ki for SAM [3].

*MAT1A* gene down-regulation at the transcriptional level, in alcoholic hepatitis, cirrhosis and HCC [4], largely depends on *MAT1A* promoter methylation and histone H4 deacetylation and on *MAT1A* mRNA interaction with AUF1 protein, which enhances its decay [5–8]. On the contrary, *MAT2A* gene is up-regulated in HCC due to the hypomethylation of its promoter and histone H4 acetylation, and interaction of *MAT2A* mRNA with the HuR protein, which increases its stability [5–8]. This molecular event (MAT1A/MAT2A switch) is responsible for the decrease in SAM/SAH (S-adenosylhomocysteine) ratio in cirrhosis and HCC. Various trans-activating factors such as Sp1, c-Myb (avian myeloblastosis viral oncogene homolog), NF-kB (nuclear factor kappa-b), and AP-1 participate in *MAT2A* transcriptional up-regulation in HCC [9].

Reduced *MAT1A* expression in HCC has also been attributed to miRNAs up-regulation [10]. MiR-664, miR-485-3p, and miR-495 individually induce *MAT1A* expression in liver tumor cells. Stable miRNAs-664/485-3p/495 overexpression in these cells increases hepatocarcinogenesis in an orthotropic liver cancer model in nude mice [10]. Also, miRNAs-664/485-3p/495 knockdown reduces liver carcinogenesis and lung metastases in nude mice parenchymally injected with HepG2 cells [10]. Recent research showed that miR-21-3p reduces the expression of *MAT2A* and *MAT2B* in HepG2 cells, by targeting their 3’-UTRs [11].

MiR-203 suppresses the proliferation and migration and promotes apoptosis of lung cancer cells through c-*SRC* and *LIN28B* inhibition [12, 13]. By targeting *RUNX2* (runt-related transcription factor 2), miR-203 and miR-135 impair the progression of breast cancer [14]. MiR-203 also inhibits prostatic cancer metastatic potential by suppressing RAP1A (ras-related protein 1A) expression [15] and suppresses *ZNF217* (zinc finger protein 217) oncogenic activity in colorectal cancer [16]. miR-203 also plays a role in HCC development and progression [17], enhances apoptosis of HCC cells by regulating *EZH2* (enhancer of zeste, drosophila, homolog 2) and *BMI-1* (leukemia viral bmi-1 oncogene, mouse, homolog of) [18], and inhibits HCC cell proliferation by targeting *SURVIVIN* [19], *RASAL2* (ras protein activator-like 2) [20], *ADAM9* oncogene and the HULC pro-tumorigenic long non-coding RNA [21] and the long non-coding RNA DLX6-AS1 and *MMP2* (matrix metalloproteinase 2) [22]. The administration of recombinant miR-203 adenovirus in a rat model with liver cirrhosis and diffused HCC, followed by partial hepatectomy, inhibits proliferation and lung metastasis of the residual HCC [23].

In most of these studies [17–21, 23] miR-203 targets were not identified or the effects of miR-203 on supposed targets were not proven. Therefore, it cannot be excluded that some of the observed miR-203 effects were indirect, induced by yet unidentified genes. Nevertheless, the identification of functionally important target genes of a specific miRNA and recognition of its mechanisms of action is essential for understanding its biological function. We identified, by bioinformatics analysis, using the TargetScan and the miRanda algorithms as sequence-based miRNA target prediction softwares, *MAT2A* and *MAT2B* genes as putative targets of miR-203. In recent years, the alterations of *MAT1A, MAT2A* and *MAT2B* activity and expression along hepatocarcinogenesis were the object of several researches [24]. The study of miRNAs impacting on these genes is important, due to the central role of methionine cycle in HCC pathogenesis [3, 7]. In the present study, we analyzed the behavior of the three MATs and miR-203 in HCC with different prognosis and in liver tumor cell lines, explored the functional effects of *MAT2A* and *MAT2B* targeting by miR-203, and evaluated the *MAT2B* oncogenic role in HCC.

## RESULTS

### Association of MAT2A and MAT2B overexpression with poor HCC prognosis

Previous work in our laboratory identified and analyzed HCC subgroups with poorer prognosis (survival < 3 years after partial liver resection; HCCP) and better prognosis (survival > 3 years; HCCB) showing lower decrease in *MAT1A* expression and lower increase in *MAT2A* expression in HCCB [7, 8]. Two groups of 13 patients with HCCB and 13 patients with HCCP were used to evaluate MATs expression (Table 1). No significant differences between the two groups occurred as concerns patients’ sex, etiology, and presence of cirrhosis and Edmondson-Steiner grade. Significantly bigger tumor size and higher alpha-fetoprotein secretion were found in HCCP than in HCCB.

**Table 1:** Clinicopathological features of HCC patients

	HCCB^a^	HCCP^b^
No of patients		
Male	12	13
Female	1	0
Age (mean ± SD)	64.2 ± 5.3	69.1 ± 3.3
Etiology		
HBV	8	10
HCV	3	3
Ethanol	1	0
Cirrhosis		
+	12	11
–	1	2
Tumor size^c^		
>5 cm	2	12
<5 cm	11	0
Edmonston and Steiner grade		
I	3	0
II	7	4
III	3	6
IV	0	3
Alpha fetoprotein secretion^d^		
>300 ng/ml of serum	5	12
<300 ng/ml of serum	8	1
Survival after PH (months). Mean ± SD^e^	62 ± 9	18 ± 9

^a^HCCB, HCC with better prognosis (survival > 3 years).

^b^HCCP, HCC with poorer prognosis (survival < 3 years).

^c^Fisher Exact Text, *p* = 0.03.

^d^Fisher Exact Text, *p* = 0.01.

^e^*p* < 0.0001.

**Figure 1:**
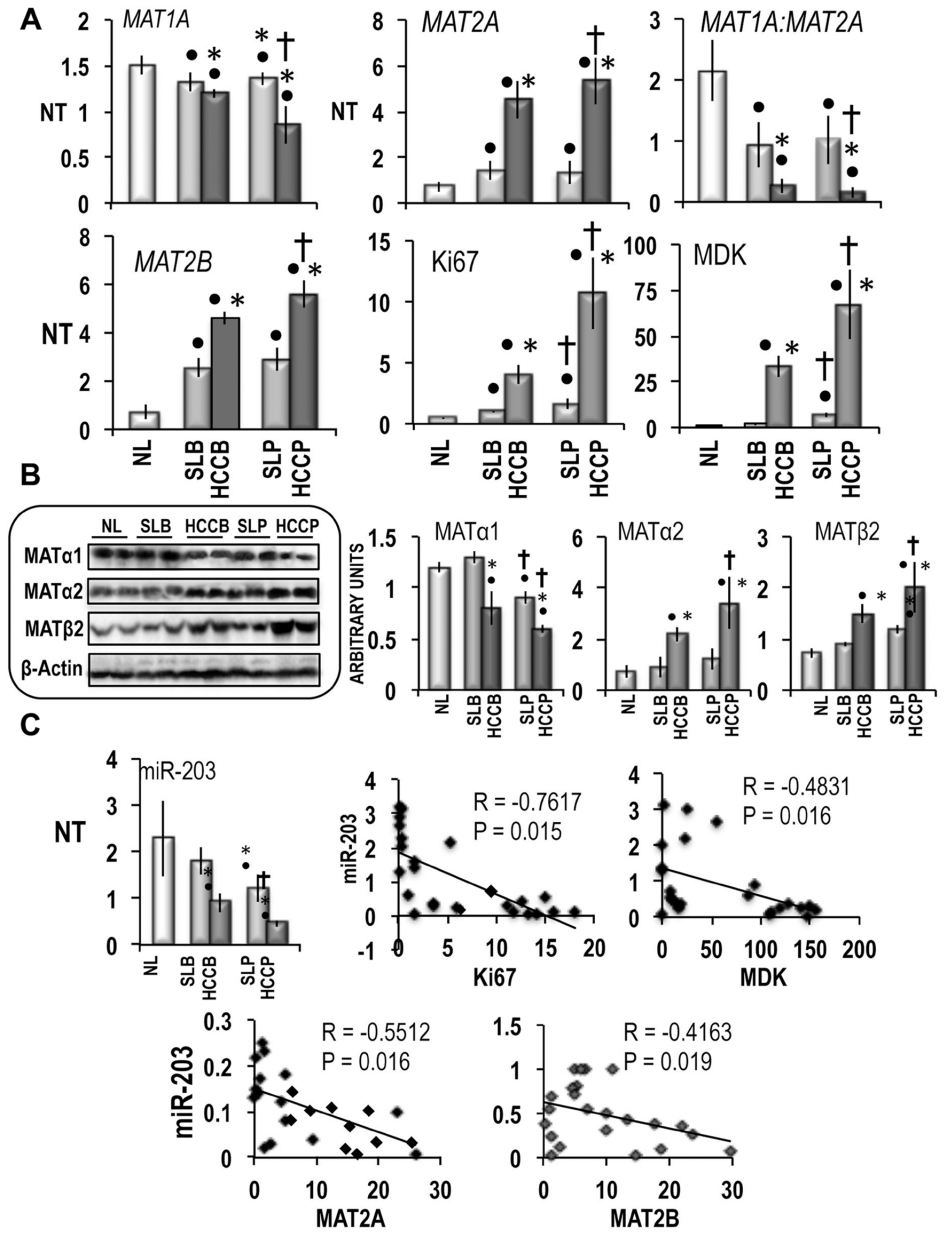
*MAT1A, MAT2A, MAT2B* and miR-203 expression in human HCC with different prognosis (**A**) *MAT1A, MAT2A*, *MAT2B, Ki67* and *MDK* gene expression. The results are expressed as N-fold differences in target gene expression relative to the RNR-18 expression, named N Target (NT). NT = 2^–ΔCT^, ΔCT of each sample was calculated by subtracting the Ct of the target gene from the Ct of the RNR-18 gene. (**B**) Representative Western Blot of MATα1, MATα2, and MATβ2, in HCCs with different prognosis and correspondent surroundings. Chemiluminescence analysis: optical densities were normalized to β-actin levels and expressed in arbitrary units. (**C**) miR-203 expression (NT = 2^–ΔCT^) and Spearman’s correlation analysis of miR-203. A total of 26 cases (13 HCCB and 13 HCCP) were used for the correlation analysis. Data are means ± standard deviation (SD) of six experiments. Mann-Whitney test: Point, different from NL for *P* < 0.001. Asterisk, different from SL for at least *P* < 0.01. Dagger, HCCP/SLP different from HCCB/SLB for *P* < 0.001. Abbreviations: HCCB, HCC with better prognosis (survival > 3 years) and correspondent surrounding (SLB); HCCP, HCC with poorer prognosis (survival < 3 years) and corresponding surrounding (SLP).

The results in Figure 1A show a decrease in *MAT1A* mRNA levels in HCCs, compared to normal livers, with lowest levels in HCCP. The decrease in *MAT1A* expression was associated with a sharp increase in *MAT2A* mRNA levels both in HCCB and HCCP, with highest values in HCCP. Much lower changes of *MAT1A* expression occurred in surrounding livers, with no significant difference between SLB and SLP. Because of these changes, *MAT1A:MAT2A* ratio showed great decreases in SLs and HCCs with lowest values in HCCP. *MAT2B* mRNA levels underwent alterations close similar to those of *MAT2A*, the highest values being found in HCCP. The latter tumors exhibited the highest expression of *Ki67* and *MDK*, markers of HCC proliferation and poor differentiation [25, 26], respectively.

Western blot analysis confirmed these results (Figure 1B), showing a significant decline of MATα1 protein in SLP and HCCs, with lowest MATα1 levels in HCCP, and significant increases of MATα2 and MATβ2, in HCCB and HCCP, with highest values in HCCP. As concerns non-neoplastic surrounding liver, significant rise of MATβ2 was only found in SLP.

The expression of miR-203 exhibited significantly lower values in SLP and HCC than in normal liver, with HCC levels lower than surrounding levels, and SLP/HCCP values lower than SLB/HCCB values (Figure 1C). Furthermore, miR-203 was negatively correlated with *Ki67, MDK, MAT2A* and *MAT2B* expression in HCCs (Figure 1C).

**Figure 2:**
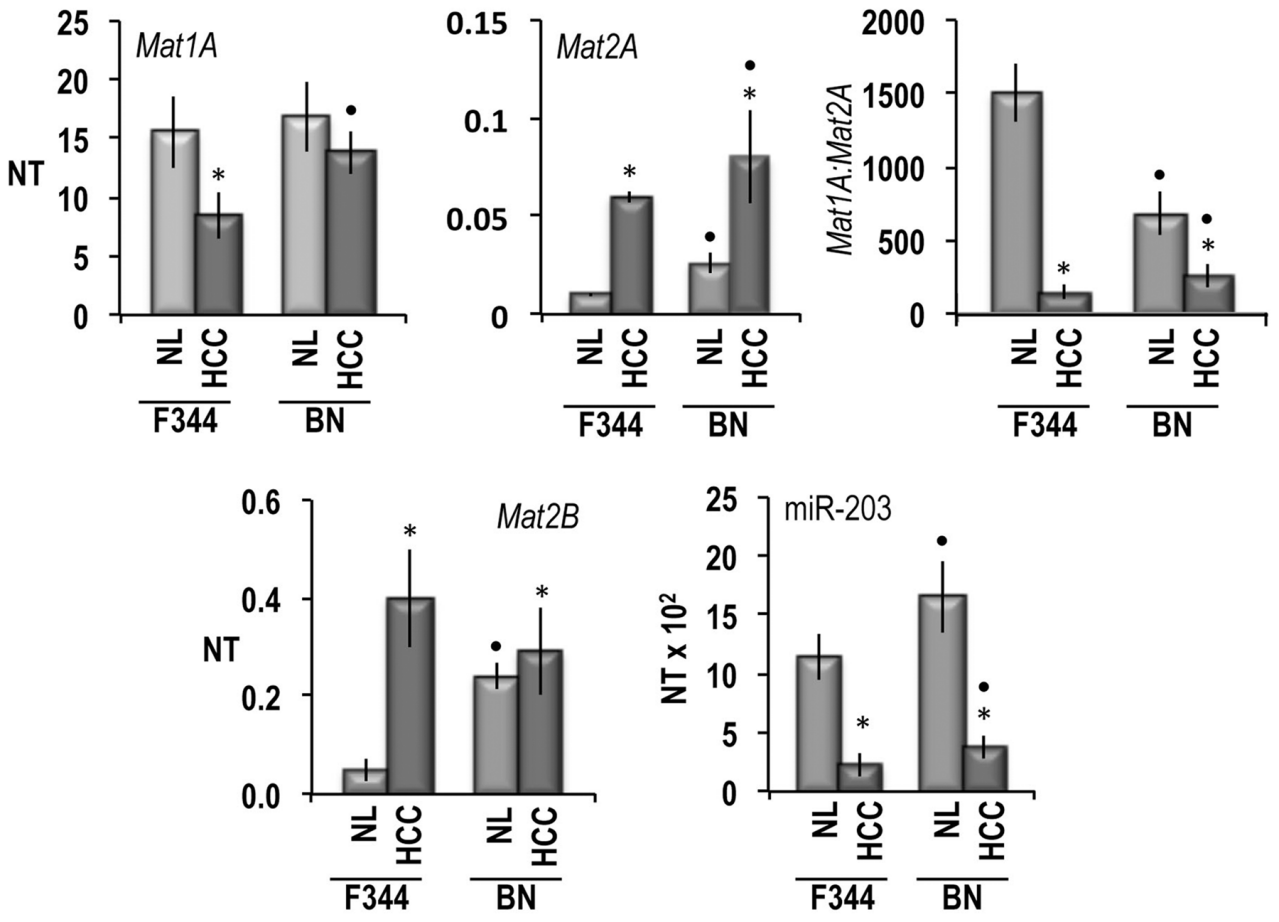
Mat1A, Mat2A, Mat2B, and mR-203 expression, in normal liver (NL) and HCCs of F344 and BN rats The results are expressed as N-fold differences in target gene expression relative to the RNR-18 expression, named N Target (NT). NT = 2^–ΔCT^, ΔCT of each sample was calculated by subtracting the Ct of the target gene from the Ct of the RNR-18 gene. Data are means ± standard deviation (SD) of six experiments. Tukey-Kramer test: asterisk: differences from control, at least *P* < 0.05. Point: F344 versus BN: at least *P* < 0.05.

Many alterations of metabolic and signaling pathways, including the methionine cycle are under genetic control in HCC [27]. To evaluate the genetic control of Mats and miR-203 expression, the latter was comparatively determined in HCC induced in F344 and BN rats, genetically susceptible and resistant to hepatocarcinogenesis, respectively [27]. As shown in the Figure 2, the *Mat1A* mRNA level decreased and the *Mat2A* and *Mat2B* mRNAs levels increased in HCC of F344 rats with respect to normal liver values, while they underwent very low change in HCC of BN rats. In the latter rats, normal liver exhibited significantly higher levels of *Mat2A* and *Mat2B* mRNAs than in F344 rats. As a consequence of the changes of *Mat1A* and *Mat2A* expression, the *Mat1A:Mat2A* ratio underwent great decreases in HCCs of F344 rats and a significantly lower decrease in BN HCCs. MiR-203 expression was significantly higher in normal liver of BN than F344 rats. It sharply decreased in HCCs of both strains, remaining significantly higher in BN HCCs than in F344 HCCs.

### MiR-203 inhibits the expression of MAT2A and MAT2B in liver cancer cells

**Figure 3:**
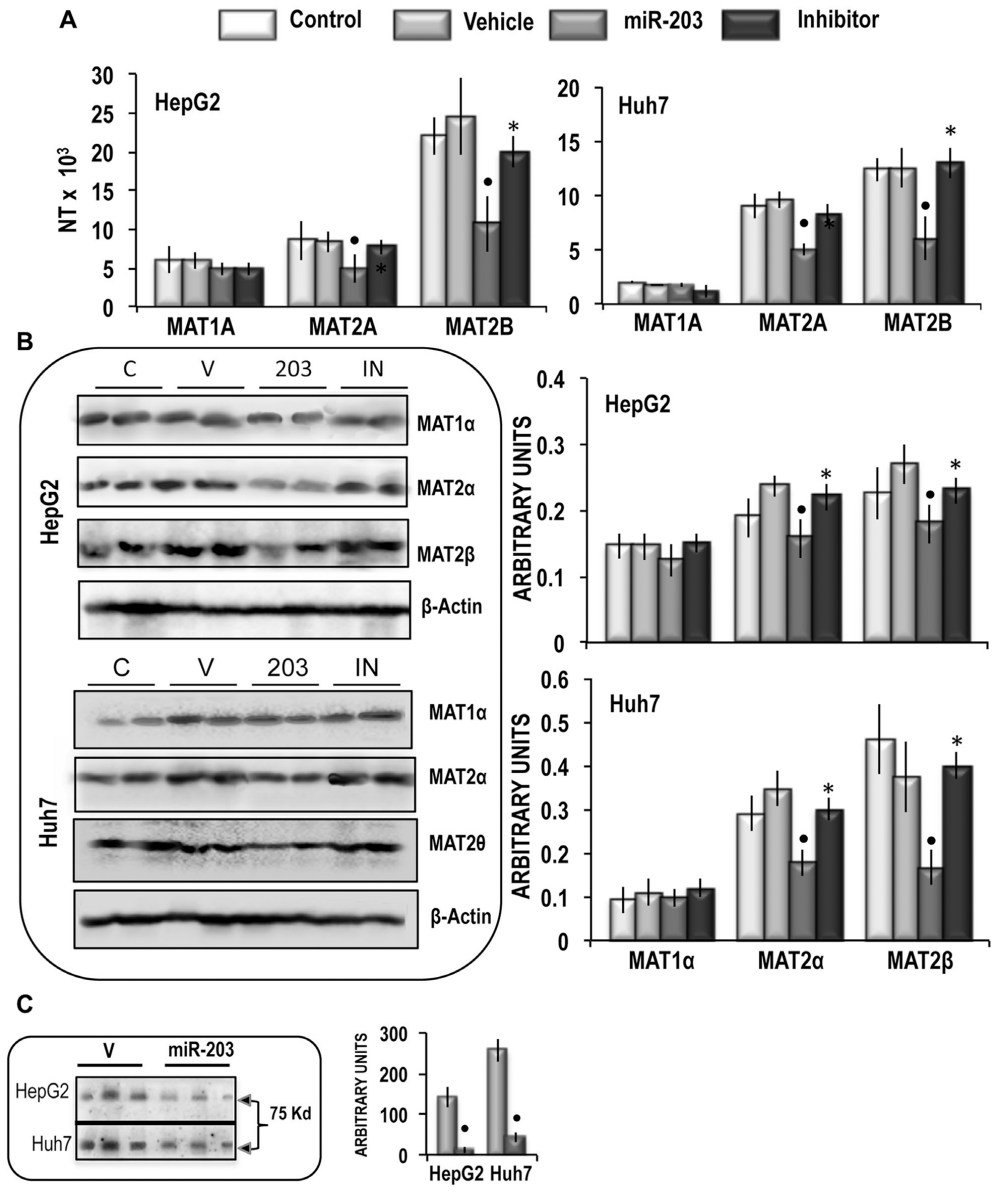
Analysis of the effects of the forced expression of miR-203 on MATs mRNA and protein expression, MATαβ2 complex, and SAM content in HepG2 and Huh7 cells (**A**) *MAT1A, MAT2A*, *MAT2B* gene expression. The results are expressed as N-fold differences in target gene expression relative to the RNR-18 expression, named N Target (NT). NT = 2^–ΔCT^, ΔCT of each sample was calculated by subtracting the Ct of the target gene from the Ct of the RNR-18 gene. Data are means ± standard deviation (SD) of 5 experiments. (**B**) Representative Western Blot of MAT1α, MAT2α, and MAT2β. Chemiluminescence analysis: optical densities were normalized to β-actin levels and expressed in arbitrary units. Data are means ± standard deviation (SD) of 5 experiments. Mann-Whitney test: Point, different from V (vehicle) for *P* < 0.001. Asterisk, different from miR-203 for *P* < 0.001. (**C**) Immunoprecipitation analysis. The presence of MAT2β was evaluated in protein extracts immunoprecipitated by anti-MAT2α antibodies. Data are means ± SD of 3 experiments. Mann-Whitney test: Point, different from V (vehicle) for *P* < 0.001.

**Figure 4:**
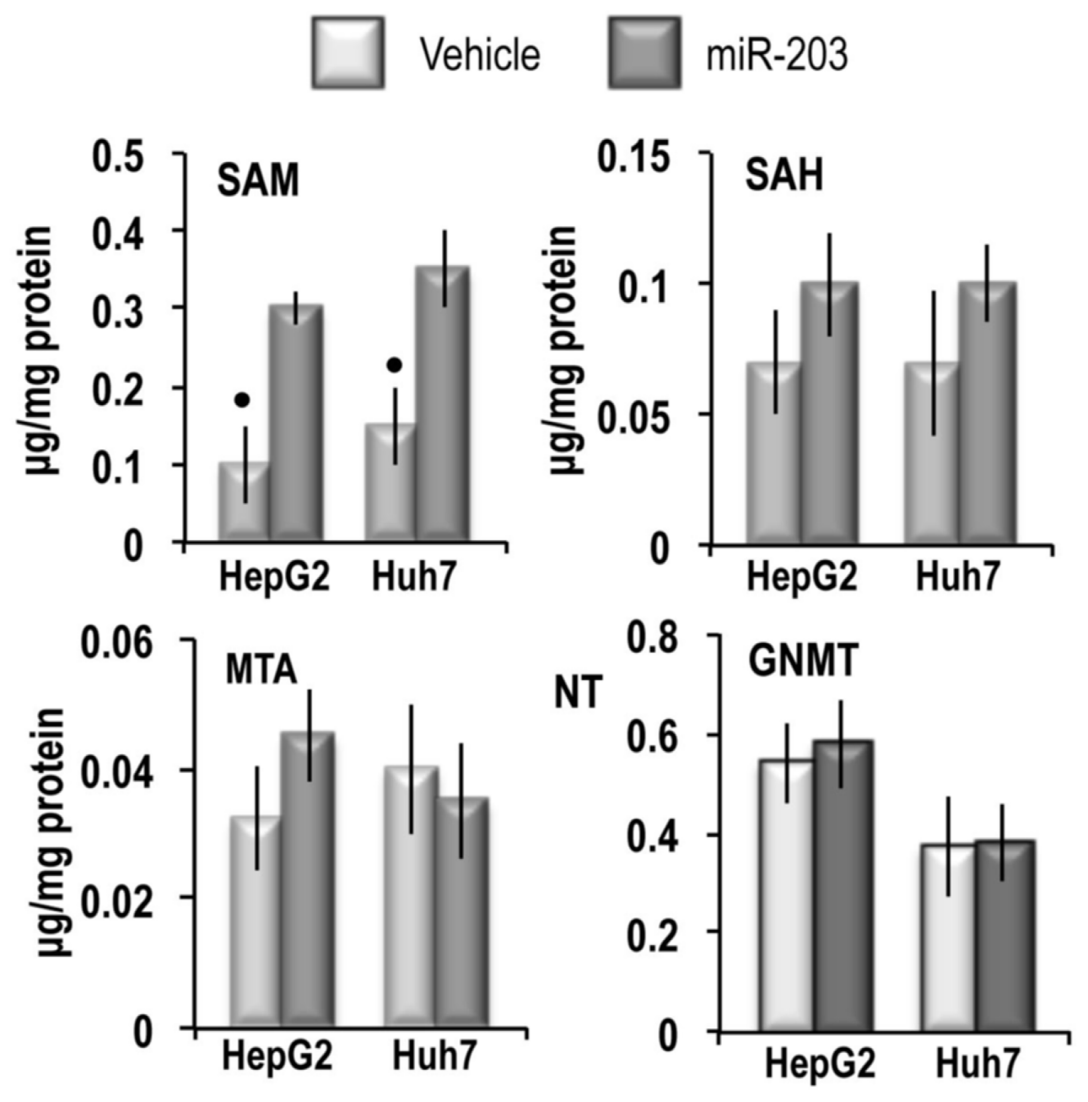
SAM, SAH and MTA content and GNMT expression in HepG2 and Huh7 cell lines For the quantitative analysis of SAM, SAH, and MTA the areas of the chromatographic peaks in cells extracts was compared with that of standard solutions. Data are means ± SD of 3 experiments. Mann-Whitney test: Point, different from V (vehicle) for *P* < 0.001. The results of the analysis of GNMT expression are expressed as N-fold differences in target gene expression relative to the RNR-18 expression, named N Target (NT). NT = 2^–ΔCT^, ΔCT of each sample was calculated by subtracting the Ct of the target gene from the Ct of the RNR-18 gene. Data are means ± standard deviation (SD) of 3 experiments.

To assess the effect of miR-203 on *MAT2A* and *MAT2B,* HepG2 and Huh7 liver cancer cells were transfected with hsa-miR-203-3p. These experiments revealed that 48 h after miR-203 transfection, *MAT2A* and *MAT2B* mRNA expression (Figure 3A) and MATα2 and MATβ2 protein expression (Figure 3B) were sharply inhibited. This inhibition was abolished by the miR-203 inhibitor. No changes of *MAT1A* expression occurred. Immune-precipitation analysis (Figure 3C) showed that miR-203 reduced by 96% and 82% the complex MATα2/MATβ2 in HepG2 and Huh7 cells, respectively. These changes were associated with ∼33% and 35% increases in SAM content in HepG2 and Huh7 cells, respectively, whereas the SAH and MTA contents were not significantly modified (Figure 4). In addition, no changes in *GNMT* expression, another known regulator of SAM level [28], were detected in miR-203 treated cells (Figure 4). Furthermore, miR-203 inhibited *BIRC5* (SURVIVIN) and *RASAL2* expression (Supplementary Figure 1), thus confirming its suppressor activity for HCC [19, 20].

### MAT2A and MAT2B 3’-UTR contain functional binding sites for miR-203

**Figure 5:**
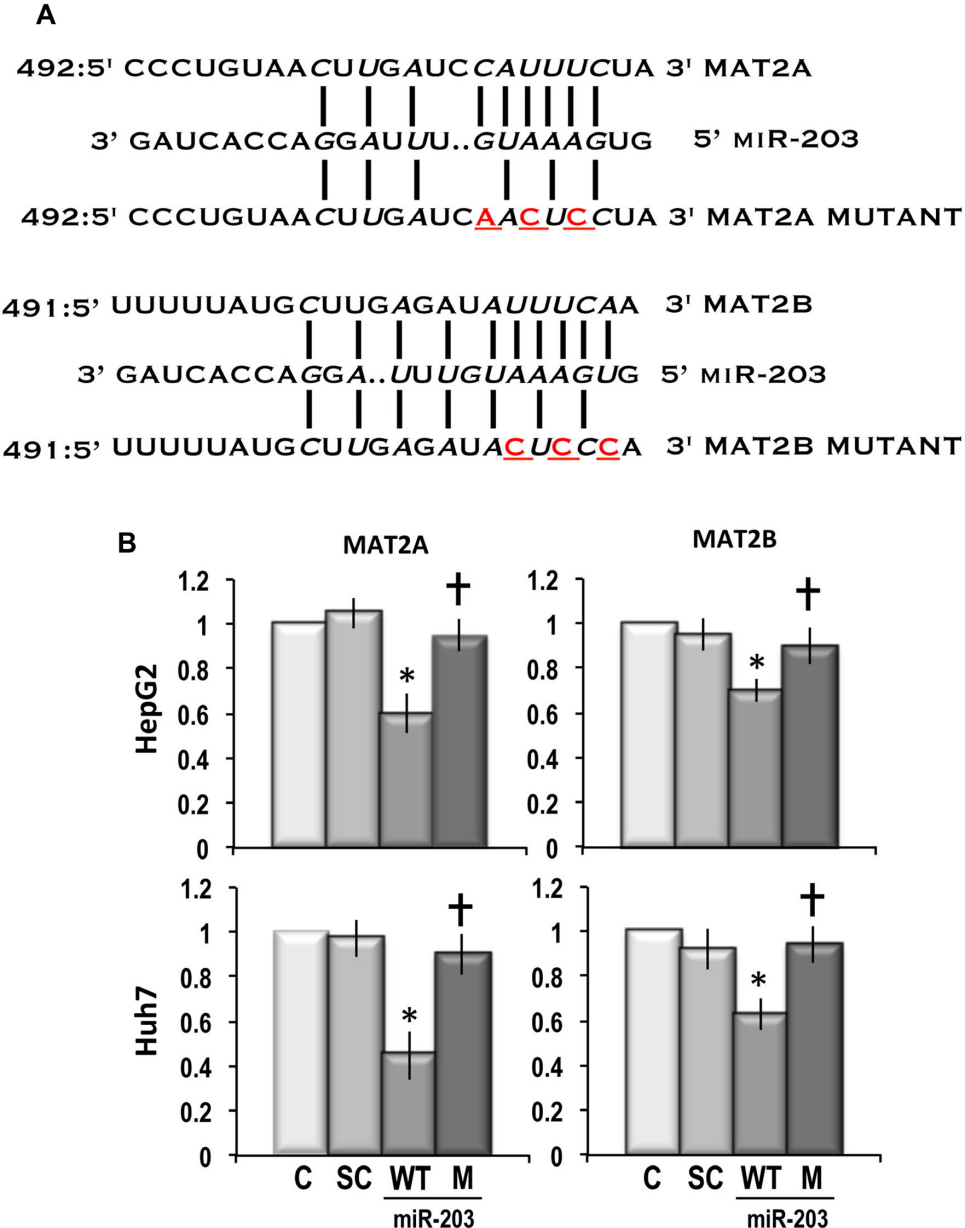
Dual luciferase reporter assay showing that *MAT2A* and *MAT2B* are direct targets of miR-203 (**A**) Schematic representation of the putative 3’-UTR binding sites of *MAT2A* and *MAT2B* for miR-203. (**B**) The relative luciferase activity of cells was measured on HepG2 and Huh7 cells. Cells were transfected with either wild-type-UTR-reporter or mutant UTR-reporter (300 ng), the control Renilla luciferase reporter plasmid pRL-TK (10 ng), and 90 nM of hsa-miR-203 mimic. Data are means ± SD of 3 independent experiments. Abbreviations: C, control; SC, scramble; WT and M, wild-type-UTR-reporter and mutant UTR-reporter. Mann-Whitney test: Point, different from control for *P* < 0.001. Dagger, different from MAT2A/MAT2B 3’-UTR for *P* < 0.001.

Figure 5A shows that 3’-UTRs of *MAT2A* and *MAT2B* contain putative binding sites for miR-203. To determine if *MAT2A* and *MAT2B* are directly targeted by miR-203, full-length 3’-UTRs of *MAT2A* and *MAT2B* were separately cloned into luciferase reporter vectors and the dual luciferase reporter assay system was used to quantify the reporter activity in transfected HepG2 and Huh7 cell lines. As shown in Figure 5B, miR-203 inhibited by ∼40% and 52% the expression of the luciferase reporter containing *MAT2A* 3’-UTR, in HepG2 and Huh7 cells, respectively. In these cell lines, the expression of the luciferase reporter containing *MAT2B* was inhibited ∼30% and 38% by miR-203, respectively. These effects were totally absent when scramble miRNA was used. They were also almost totally absent when the luciferase reporter containing mutated *MAT2A* or *MAT2B* 3’-UTR was tested in the presence of miR-203.

### Functional effects of miR-203 in liver tumor cell lines

**Figure 6:**
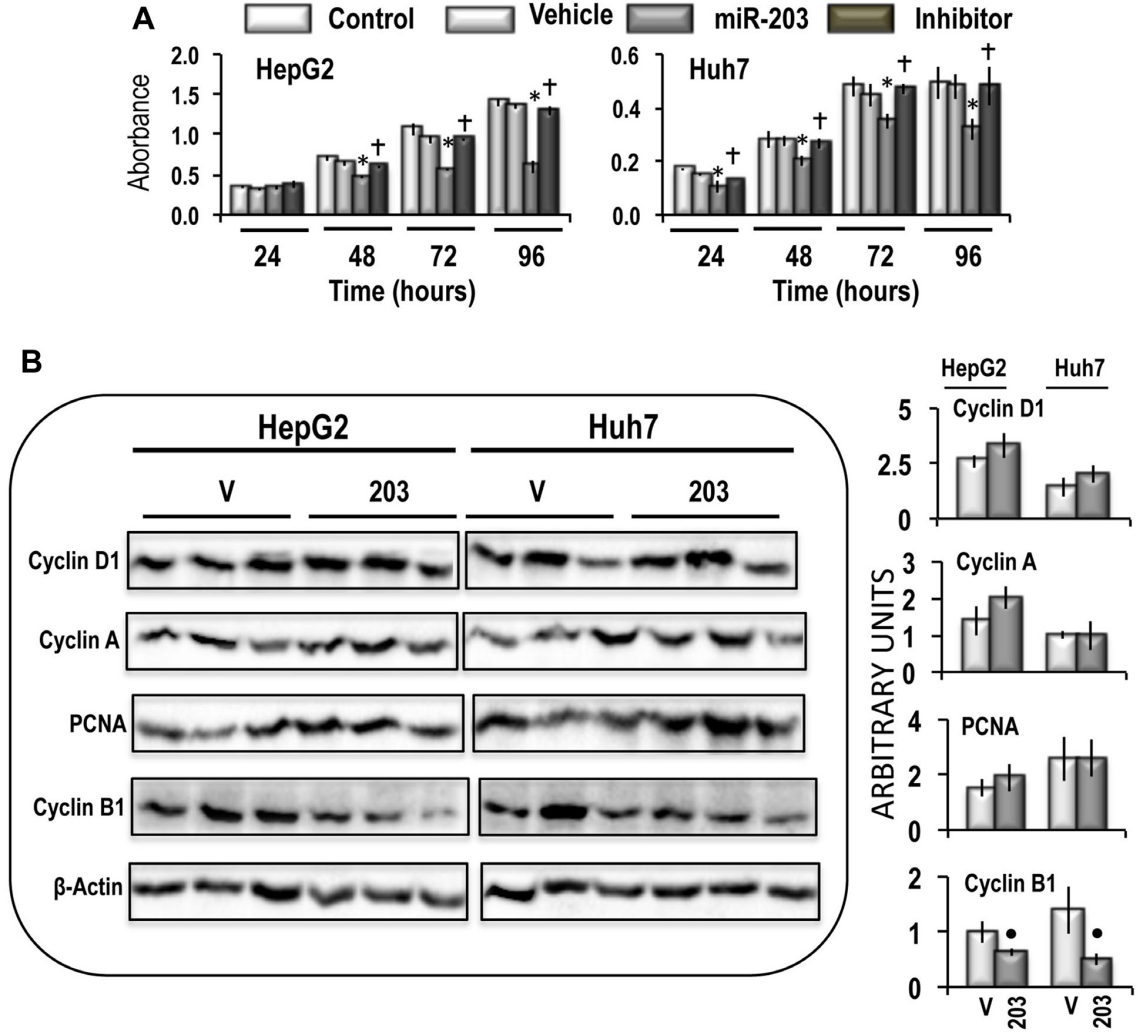
Effect of miR-203 forced expression on the viability, and expression of Cyclin D1, Cyclin A, PCNA, and Cyclin B1 in HepG2 and Huh7 cells (**A**) MTT evaluation of cell viability at different time after seeding. Data are means (SD) of 5 experiments. Mann-Whitney test: Asterisk, different from vehicle (V) for *P* < 0.001. Dagger, different from miR-203 for *P* < 0.001. (**B**) Representative Western Blot of Cyclin D1, Cyclin A, PCNA, and Cyclin B1. Chemiluminescence analysis: optical densities were normalized to β-actin levels and expressed in arbitrary units. Data are means ± standard deviation (SD) of 3 experiments. Mann-Whitney test: Point, different from V (vehicle) for *P* < 0.001.

MiR-203 transfection in HepG2 and Huh7 cells induced a significant restraint in cell growth, between 48 and 96 hours after transplantation, which was completely suppressed by the miR-203 inhibitor (Figure 6A). Subsequently, the analysis of the levels of Cyclin D1, a marker of G1 phase [29], Cyclin A2 and PCNA, markers of S phase [30, 31], and Cyclin B1, a marker of G2 phase [32] was employed. No significant effects of miR-203 manipulation on Cyclins D1, Cyclin A2, and PCNA were observed, whereas miR-203 forced overexpression induced a significant decrease of Cyclin B1 level in both cell lines, thus suggesting a block of S/G2 transition of the cell cycle (Figure 6B).

**Figure 7:**
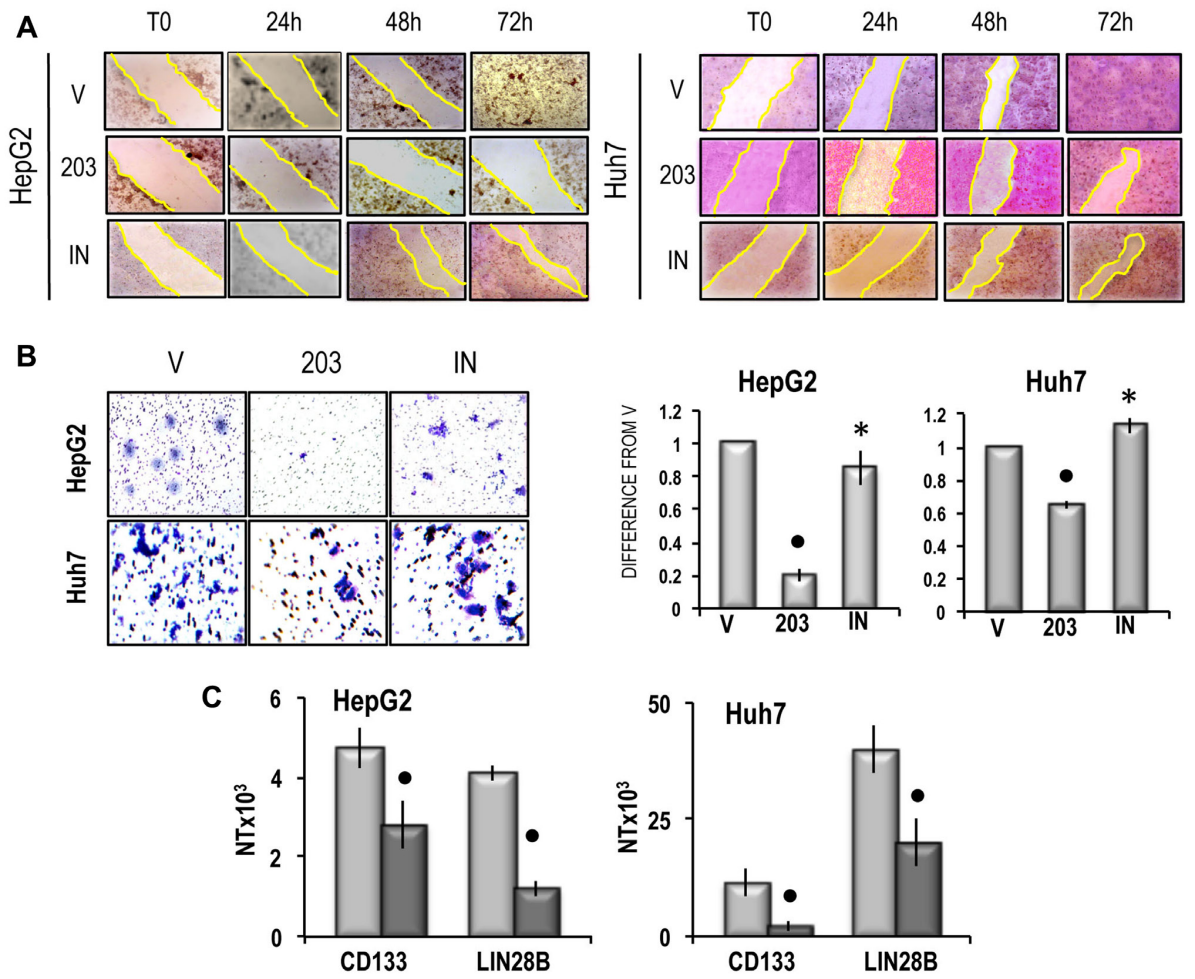
(**A**) Representative images of migration ability of cells transfected with miR-203 in RNAiMAX vehicle (V) or miR-203 inhibitor (IN) evaluated by wound healing assay. The restriction of the wounded area was evaluated at the times indicated after wounding (zero-time). Three independent analyses of cell migration *in vitro* did not show significant variations of the wounded area restriction at the different times. (**B**) Left panels: representative images of invasivity of cells transfected with miR-203 in RNAiMAX vehicle, empty vehicle (V) or miR-203 inhibitor (IN); rigth panels: mean differences from V ± SD (*n* = 3). Mann-Whitney test: Point, different from V for *P* < 0.001; asterisk, different from miR-203 for *P* < 0.001. (**C**) *CD133* and *LIN28B* expression. The results are expressed as N-fold differences in target gene expression relative to the RNR-18 expression, named N Target (NT). NT = 2^–ΔCT^, ΔCT of each sample was calculated by subtracting the Ct of the target gene from the Ct of the RNR-18 gene. Data are means ± standard deviation (SD) of 5 experiments. Mann-Whitney test: Point, different from V for *P* < 0.001.

**Figure 8:**
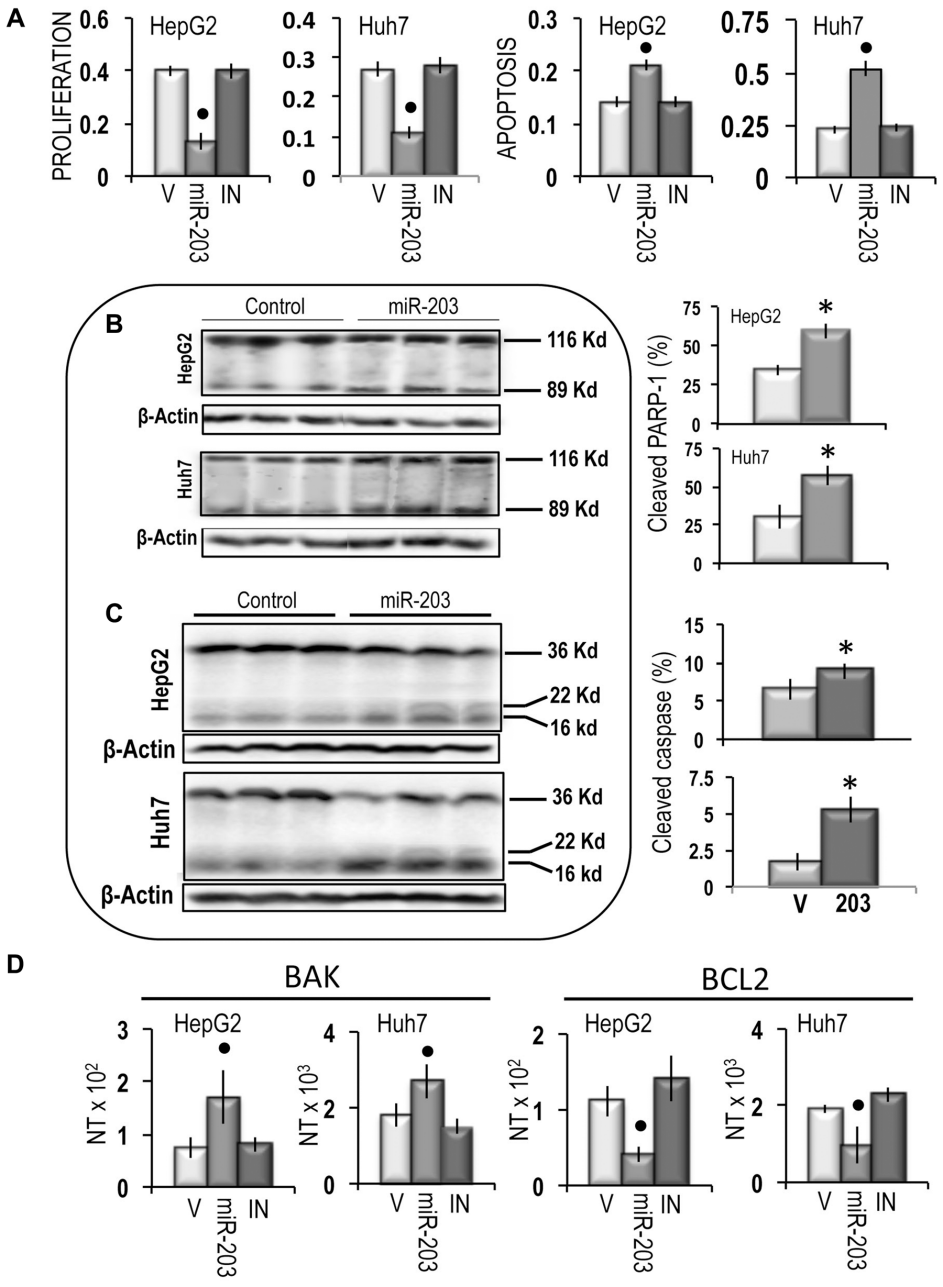
Apoptogenic effect of miR-203 on HepG2 and Huh7 cells **(A)** Evaluation of cell proliferation and cell death by the Cell Death Detection Elisa Plus Kit. Data are means (SD) of 3 experiments. Mann-Whitney test: Point, different from vehicle (V) for *P* < 0.001 **(B)** Representative Western blots of PARP-1 cleavage. **(C)** Representative Western blots of Caspase 3 cleavage. Chemiluminescence analysis: optical densities were normalized to β-actin levels and expressed in arbitrary units. Data are means (SD) of 5 experiments. Mann-Whitney test: Asterisk, different from V for *P* < 0.001. **(D)** Expression of *BAK* and *BCL2* genes. The results are expressed as N-fold differences in target gene expression relative to the RNR-18 expression, named N Target (NT). NT = 2^–ΔCT^, ΔCT of each sample was calculated by subtracting the Ct of the target gene from the Ct of the RNR-18 gene. Data are means (SD) of 4 experiments. Mann-Whitney test: Point, different from V for *P* < 0.001.

The growth inhibitory effect of miR-203 was paralleled by a sharp inhibition of cell migration in both cell lines, which was partially prevented by the miR-203 inhibitor (Figure 7A). An inhibition of about 80% and 38% of cell invasion was also induced by miR-203 in HepG2 and Huh7 cells, respectively, which was prevented by the miR-203 inhibitor (Figure 7B). Furthermore, miR-203 caused a significant restraint of the expression of *CD133* and *LIN28B*, two genes associated with cancer cells stemness [33, 34], both in HepG2 and Huh7 cells (Figure 7C). Proliferation analysis confirmed the inhibition of cell proliferation in miR-203 transfected HepG2 and Huh7 cell lines and showed a significant increase in apoptosis (Figure 8A), that was confirmed by the rise in PARP-1 and Caspase 3 cleavage (Figure 8B and 8C). Furthermore, the cell death stimulus induced by miR-203 overexpression was associated with a significant decrease in the expression of the anti-apoptotic *BCL2* gene and the upregulation of the pro-apoptotic *BAK* gene (Figure 8D).

### Oncogenic activity of MAT2B

**Figure 9:**
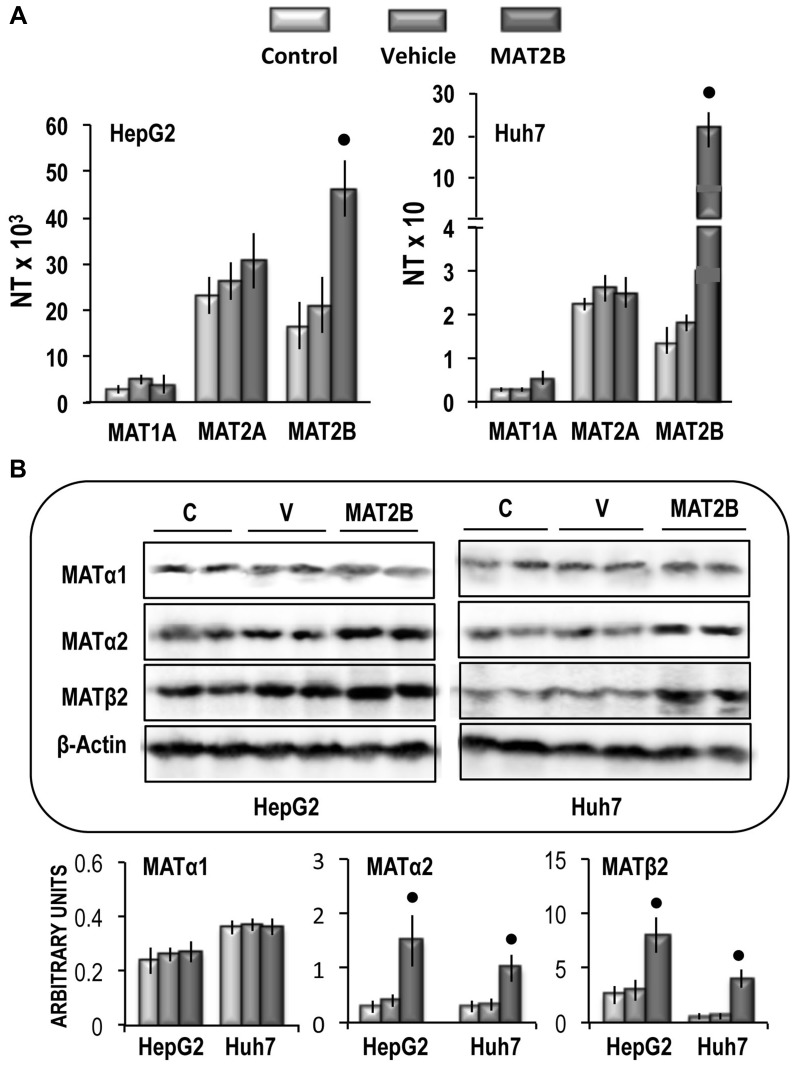
**(A)** *MAT1A, MAT2A* and *MAT2B* gene expression in HepG2 and Huh7 cells. The results are expressed as N-fold differences in target gene expression relative to the RNR-18 expression, named N Target (NT). NT = 2^–ΔCT^, ΔCT of each sample was calculated by subtracting the Ct of the target gene from the Ct of the RNR-18 gene. **(B)** Representative Western Blot of MAT1α, MAT2α, and MAT2β. Chemiluminescence analysis: optical densities were normalized to β-actin levels and expressed in arbitrary units. Data are means ± standard deviation (SD) of 5 experiments. Point, different from V for *P* < 0.001.

Previous work has demonstrated that shRNA interference targeting *MAT2B* can induce cell apoptosis and growth-inhibition in HCC cell lines [35]. This suggests a possible oncogenic effect of *MAT2B*. To test this possibility, liver cancer cell lines were transiently transfected with *MAT2B*. This induced a ∼2.2-fold and 15-fold increases in *MAT2B* mRNA expression in HepG2 and Huh7 cells, respectively, without changes of *MAT1*A and *MAT2A* mRNA expression (Figure 9A). A different behavior occurred at the protein level: *MAT2B* transfection induced ∼2.7 and 8.7 fold increases in MATβ2 protein expression, and ∼4 and 2.8 fold increases in MATα2 protein expression in HepG2 and Huh7 cells, respectively (Figure 9B).

**Figure 10:**
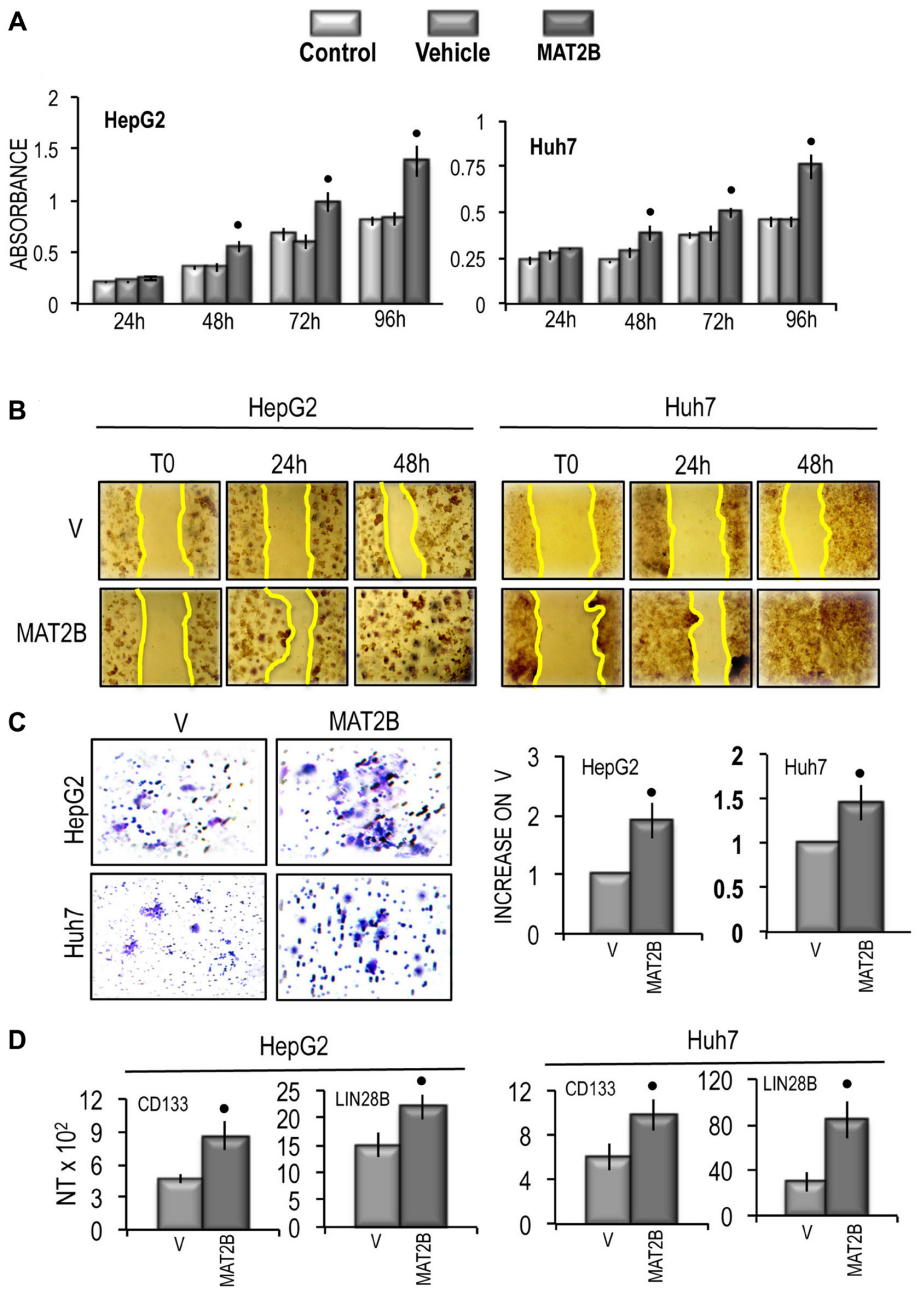
Effect of MAT2B transfection on HepG2 and Huh7 cell viability, cell migration and cell invasivity *in vitro* and of the expression of CD133 and LIN28B **(A)** MTT evaluation at different times after seeding. Data are means of 5 experiments. Mann-Whitney test: Point, different from vehicle (V) for *P* < 0.001. **(B)** Representative images of the migration of cells transfected with empty vehicle (V) or MAT2B in PCMV6 vehicle or evaluated by wound healing assay. The restriction of the wounded area was evaluated at the times indicated after wounding (zero-time). **(C)** Left panels: representative images of migration ability of cells transfected with empty vehicle (V) or MAT2B in PCMV6 vehicle. Right panels: mean differences from V ± SD (*n* = 3). Mann-Whitney test: Point, different from vehicle (V) for *P* < 0.001. **(D)** CD133 and LIN28B gene expression. The results are expressed as N-fold differences in target gene expression relative to the RNR-18 expression, named N Target (NT). NT = 2^–ΔCT^, ΔCT of each sample was calculated by subtracting the Ct of the target gene from the Ct of the RNR-18 gene. Data are means ± standard deviation (SD) of 3 experiments. Mann-Whitney test: Point, different from vehicle (V) for *P* < 0.001.

**Figure 11:**
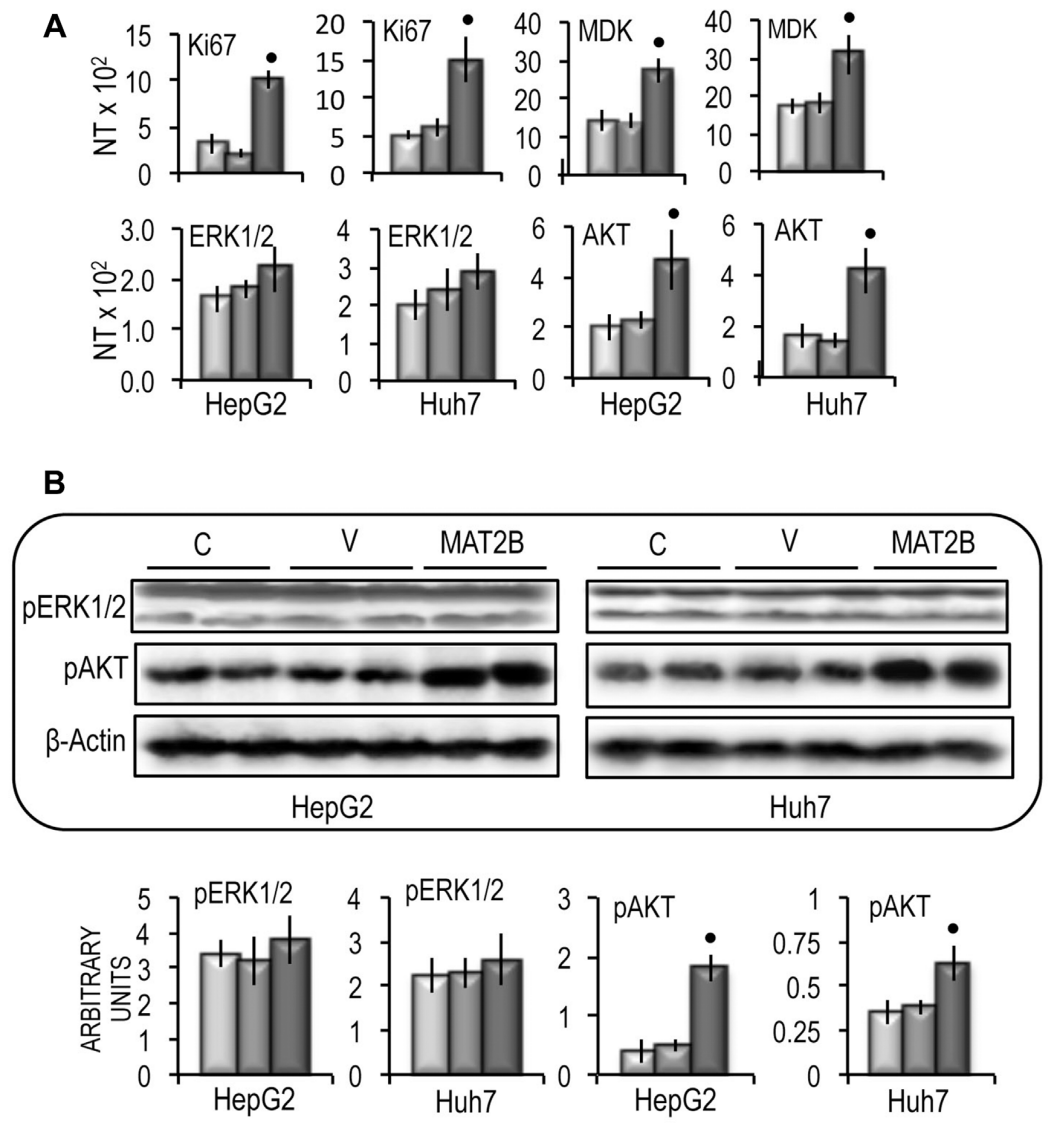
Effect of MAT2B forced expression on Ki67, MDK, ERK1/2 and AKT expression in HepG2 and Huh7 cells **(A)** Ki67, MDK, ERK1/2 and AKT gene expression. The results are expressed as N-fold differences in target gene expression relative to the RNR-18 expression, named N Target (NT). NT = 2^–ΔCT^, ΔCT of each sample was calculated by subtracting the Ct of the target gene from the Ct of the RNR-18 gene. **(B)** Representative Western Blot of pERK1/2 and pAKT(ser473). Chemiluminescence analysis: optical densities were normalized to β-actin levels and expressed in arbitrary units. Data are means ± standard deviation of 5 experiments. Mann-Whitney test: point, different from V for *P* < 0.001.

These changes were associated with a significant increase in cell growth rate between 48 h and 96 h of the *in vitro* culture of both cell lines (Figure 10A), a consistent rise in cell migration and invasion (Figure 10B and 10C), and the upregulation of *CD133* and *LIN28B* genes in the two cell lines (Figure 10D). Functional analysis revealed that *MAT2B* transfection induced consistent increases in the expression of *Ki67* and *MDK* (Figure 11A) as well as a rise of *AKT* mRNA and p-AKT protein expression in both cell lines, whereas no variation of ERK1\2 protein levels occurred (Figure 11B).

## DISCUSSION

A large deal of evidence supports the pathogenic role of the alterations of the methionine metabolic cycle in hepatic injury and hepatocarcinogenesis [24]. Therefore, it may be hypothesized that the correction of these alterations might be beneficial or curative. However, the attempts to correct the effects of SAM decrease during hepatocarcinogenesis, by administration of exogenous SAM, inhibited *Ha-Ras*, *Ki-Ras,* and c-*Myc* expression during experimental hepatocarcinogenesis and prevented HCC development [27, 36–38] but were not curative [38].

MicroRNAs are important liver cancer biomarkers [39] whose deregulation plays a role in HCC pathogenesis [40]. Thus, miRNA signatures may serve as biomarkers for HCC classification, prognostic risk stratification as well as for therapy [41]. MiR-203 is a cancer suppressor that interacts with different genes involved in hepatocarcinogenesis, such as *EZH2* and *BMI1* [18], *SURVIVIN* [19], *RASAL2* [20], *ADAM9* [21], and *MMP2* [22]. Here, we identified two new important targets of miR-203 in the liver. Specifically, we demonstrated that miR-203 targets the 3’-UTR of *MAT2A* and *MAT2B* genes, inhibiting their expression in liver cancer cell lines, and contributes to HCC prognosis, being more expressed in HCCs with better prognosis, and its expression being inversely correlated with HCC proliferation and aggressiveness markers.

Interestingly, our results strongly suggest that oncosuppressor miR-203 contributes to the genetic susceptibility to HCC [27] being more expressed in the liver of genetically resistant BN rats, less prone to HCC development than susceptible F344 rats [27]. Furthermore, slowly growing and progressing HCCs of BN rats exhibited a higher expression of the oncosuppressor miR-203 than F344 HCCs. To our knowledge, this is the first observation relating miRNA expression to the genetic susceptibility to HCC. In this respect, it is intriguing that rat *mir-203* gene is located on chromosome 6, where different susceptibility/resistance loci, determining genetic susceptibility to HCC, are located [27].

Our results also revealed that the transfection of miR-203 in HepG2 and Huh7 liver cancer cell lines resulted in consistent inhibition of cell viability associated with induction of apoptosis and suppression of the stemness features of these cell lines, as shown by the inhibition of cell migration and invasiveness and *CD133* and *LIN28B* stemness markers expression. Furthermore, miR-203 transfection induced an increase in the SAM content of transfected cells, probably dependent on the reduction of MAT1A/MAT2A switch, the latter being largely responsible for SAM levels restriction and increase in proliferation capacity of tumor cells [3, 8]. GNMT, a main enzyme in SAM metabolism [28], could also influence the SAM level. However, according to our results, miR-203 transfection did not influence GNMT expression in HepG2 and Huh7 cells. These findings agree with the observation that miR-203 significantly reduces the stemness properties of human primary keratinocytes by repressing the DeltaNp63 transcription factor [42].

*MAT2A* and *MAT2B* encode MATα2 and MATβ2 proteins possessing catalytic and regulatory roles, respectively. The *MAT2A* and *MAT2B* genes up-regulation in HCC strongly suggests that the MATαβ2 complex (therefore MATII isozyme) plays a significant role in the pathogenesis of this tumor. MAT1A/MAT2A switch has been involved in RAS/ERK, IKK/NF-kB, PI3K/AKT, and NF-kB signaling up-regulation, leading to increase in tumor cell proliferation, cell survival, and micro-vascularization [24]. Previous work in our laboratory showed that MAT1A/MAT2A switch is genetically determined and is connected to high genomic instability and HCC poor prognosis [7, 8]. Accordingly, *MAT2A* up-regulation confers a growth advantage to human HCC and colon cancer [43, 44] as well as leukemic cells [45], and its downregulation by specific siRNA inhibits BrdU incorporation and induces apoptosis in RKO adenocarcinoma and HepG2 hepatoblastoma cells [46]. Also, *MAT2B* downregulation induces cell apoptosis and growth-inhibition in HepG2 cells [35]. Less known are the effects of *MAT2B* up-regulation. The fact, however, that MATβ2 interacts with different proteins, including MATα2, strongly involved in hepatocarcinogenesis [47, 48], suggests its relevance in hepatocarcinogenesis. According to our results, *MAT2B* up-regulation does not influence the expression of *MAT1A* and *MAT2A* mRNAs, but significantly stimulates that of MATα2 protein. It may be hypothesized that this effect is secondary to the increase in MATα2 stability due to the reciprocal enhancement of the stability operated by MATα2 and MATβ2, with consequent rise in MATα2/β2 complex [48]. The oncogenic effect of *MAT2B* overexpression was evidenced by the sharp overexpression of *Ki67* and *MDK*, that highlights the rise in cell growth and aggressiveness [25, 26], increased cell migration and invasiveness, and upregulation of stemness markers. The mechanisms that mediate these effects remain poorly defined. Recent evidence indicates that miRNA-203 overexpression interferes with AKT in liver and gastric cancer cells [49, 50]. This envisages a possible interference of MATα2 and MATβ2 with AKT signaling. Accordingly, our results suggest the existence of interplay between *MAT2B* and the AKT signaling that could have an important pathogenic role due to the impact of AKT signaling on apoptosis, protein synthesis, and lipid and carbohydrate metabolism [51]. However, further work is necessary to further unravel the molecular mechanisms involved in the *MAT2B* oncosuppressor activity and in its interplay with the AKT pathway.

In conclusion, our results demonstrate, for the first time, that the tumor suppressor miR-203 targets the 3’-UTRs of *MAT2A* and *MAT2B* and inhibits their expression, and show that miR-203 expression is genetically regulated and strongly contributes to determine patients’ outcome. Therefore, miR-203 may be considered a putative predictor of HCC prognosis and a biomarker for patient stratification and, eventually, drug selection and efficacy. Furthermore, we found that miR-203 expression is inversely correlated with markers of HCC proliferation and aggressiveness, and strongly inhibits the growth, reduces stem-like features and causes death of HepG2 and Huh7 liver cancer cell lines. The data also demonstrated a strong oncogenic activity of *MAT2B*. The decreased activity of the latter and the rise of the MAT1A/MAT2A ratio, with consequent increase in SAM content, robustly contribute to the sharp growth restraint of HCC cells induced by miR-203. Thus, our findings indicate *MAT2A* and *MAT2B* as new putative therapeutic targets for HCC and underline the need of further work to evaluate the therapeutic potential of miR-203 mimics against HCC.

## MATERIALS AND METHODS

### Human tissue samples

Six normal livers and 26 HCC and corresponding surrounding non-tumorous livers (SLs) were used. Liver tissues were archival samples kindly provided by the Department of Surgery “Pietro Valdoni”, University of Rome “La Sapienza”, and the Division of Surgery of the Department of Medical, Surgery, and Experimental Sciences, University of Sassari. Informed patients’ consent and Institutional Review Board approval was obtained at these Departments.

### Animals and treatments

F344 and BN rats were treated according to the “resistant hepatocyte” protocol [51] consisting of a 150-mg/kg intraperitoneal dose of diethylnitrosamine followed by 15 days of feeding a 0.02% 2-acetylaminofluorene containing hyperprotein diet, with a partial hepatectomy at the midpoint of this feeding regime. Dysplastic nodules (32 weeks), and HCCs (57–60 weeks) were used. Animals received human care, and study protocols were in compliance with the National Institutes of Health guidelines for use of laboratory animals. Rats were killed by bleeding through thoracic aorta, under metedomidine anesthesia. Freshly removed livers were serially sectioned with ∼0.5 cm intervals. Dysplastic nodules macroscopically identified by their sharp grayish-white color, were scooped out from the liver, free of surrounding parenchyma (as verified by histological control). HCCs were collected from F344 and BN rats leaving out a small rim of neoplastic tissue. Only dysplastic nodules with diameter ≧0.03 cm^3^ were collected from both rat strains and split in half. One half of this material was processed for histology, histochemistry, and immunohistochemistry, and the other half was stored at –80°C. Histological (HE staining), histochemical (silver staining of reticulin) and immunohistochemical (glutamine synthase immunostaining) criteria were used, in addition to morphology, to classify liver lesions according to the published criteria [52–54], (data not shown).

### Cell lines and treatments

Certified HepG2 human hepatoblastoma and Huh7 human HCC cell lines were obtained from ATCC and seeded at a density of 1.5 × 10^4^ cells per well in 96-well culture plates in Optimem medium containing 5% FBS, at 37°C (Gibco, Thermo Fisher Scientific, Monza, Italy). When indicated, Lipofectamine RNAiMAX (Life Technologies, Monza, Italy) plus/minus hsa-miR-203 or negative-control or miRNA-203 inhibitor (90 nM) was included in the medium. 24 hours later, when needed, cells were transfected with pGL3 (80 ng) and pRL-TK (40 ng) luciferase plasmids, using Lipofectamine 2000 (Invitrogen) according to the manufacturer’s instructions to perform the 3’-UTR analysis. Hsa-miR-203-3p (mirVana miRNA mimic), hsa-miR-203-3p inhibitor (mirVana miRNA inhibitor) and negative-control were purchased from Ambion-Life Technologies (Milano, Italy).

### Proliferation, progression and viability indexes

Proliferation and progression indexes were evaluated in human HCC by determining *Ki-67* and *MDK* expression, respectively. Cell viability of liver tumor cell lines was determined by MTT test (Sigma Aldrich, Milano, Italy). Cell death was evaluated by the Cell Death Detection Elisa Plus Kit (Roche Molecular Biochemicals, Indianapolis, IN, USA) following the manufacturer’s protocol. For wound-healing assay, HepG2 and Huh7 cells were seeded into 24-well plates and cultured to confluence. Cells monolayers were then wounded with sterile pipette tips and washed with PBS. Pictures were acquired using a fluorescence microscope. Cell invasiveness was analyzed by the Cytoselect 24-well cell invasion kit (Cell Biolabs, San Diego, USA), with 300.000 cells/well.

### Quantitative Real-time RT-PCR

(A) Gene expression assays. Total RNAs (1 μg) were reverse transcribed into cDNA by High Capacity cDNA reverse transcription kit (Applied Biosystem, Thermo Fisher Scientific, Monza, Italy). cDNAs were amplified using specific Quantitect Primer Assays and quantified with Quantitect SYBER GreenPCR Kit (Qiagen Technologies, Milano, Italy), according to the manufacturer’s protocol, and normalized to the house-keeping transcript Hs-RRN18S. (B) Total RNA (1 μg) containing miRNAs was extracted using miRNAeasy kit and, after (RNase-free) DNase treatment, samples were reverse transcribed by miScript II RT kit, and then amplified using miScript Primer Assays and the miScript SYBER GreenPCR Kit (Qiagen Technologies), according to the manufacturer’s protocol. Hsa-miR-203-3p and RNU6-specific RT primers were purchased from Qiagen Technologies.

### Western blotting

Hepatic tissue samples and cell suspensions from cultured cancer cells were homogenized in lysis buffer (30 mM Tris, pH 7.5, 150 mM NaCl, 1% NP-40, 0.5% Na-deoxycholate, 0.1% SDS, 10% glycerol, and 2 mM EDTA) containing the Complete Protease Inhibitor Cocktail (Roche Molecular Biochemicals, Indianapolis, USA) and sonicated. Proteins were cleaned by binding G-sepharose beads & IgG normal control (rabbit, goat & mouse). Protein concentrations were determined with the Lowry-Folin assay (Sigma-Aldrich) using bovine serum albumin as standard. Membranes were probed with the primary antibodies shown in Table 2 and incubated with secondary antibodies, and revealed with the Super Signal West Pico (Pierce Chemical Co., New York, USA). Densities of the protein bands were normalized to β-actin levels and calculated by ImageQuant Software.

**Table 2:** List of the primary antibodies used for western blotting and immunoprecipitation analysis

Protein	Antibody	Epitope mapping
Actin	Cell Signaling Technology (cod. 4967)	Aminoterminal residues
Caspase 3	Cell Signaling Technology (cod. 96627)	Residues surrounding Asp 175
Cyclin A	Santa Cruz Biotecnology (c-19, cod. sc. 751)	C-terminus
Cyclin B1	Santa Cruz Biotecnology (H-10, cod. sc. 594)	C-terminus
Cyclin D1	Santa Cruz Biotecnology (cod. sc. 718)	C-terminus
MATα1	Abcam (cod. AB29176)	
MATα2	Abcam (cod. AB189208)	N2-terminal
MATβ2	NovusBiologicals	NBP1-82797
pAKT	Cell Signaling Technology (cod. 4051)	Residues surrounding Ser 473
PARP1	Santa Cruz Biotecnology (cod. sc. 1562)	N-terminus
PCNA	Santa Cruz Biotecnology (PC-10, cod. sc. 56)	
pERK1/2	Santa Cruz Biotecnology	Epitope corresponding to the motif Thr-Glu-Tyr

### Immune-precipitation analysis

Five hundred μg of protein from HepG2 and Huh7 cells were lysed in RIPA modified immune-precipitation buffer, containing protease and phosphatase inhibitors. Whole cell lysate was immune-precipitated with antibodies against MATα2 protein. Clean Blot IP Detector Reagent (Thermo Scientific) was used to reduce background. Immune-precipitated samples were separated via SDS-PAGE and treated with biotinylated secondary antibody against MATβ2. Immune-complexes were revealed and quantified as above. Densities of the protein bands were normalized to β-actin levels and calculated by ImageQuant Software.

### Construction of the Luciferase reporter plasmids

The full-length 3’-UTRs of *MAT2A* and *MAT2B* genes were amplified from genomic DNA of HepG2 cells by PCR. Forward primer containing a SpeI restriction site (5′-ATAACTAGTGTGTTAGCCTTTTTTCCCCAG-3′) and reverse primer with a HindIII restriction site (5′-ATAAAGCTTGCACTTTCTGCTTAGGGCAA-3′) were used to amplify the human *MAT2A* 3’-UTR. Forward primer (5′-GAAATAGTTTTGTATGAGTACTT-3′) and reverse primer (5′-TCTTCTGATGTAACATGTGATAC-3′), containing a ScaI and a Ndel restriction site, respectively, were used to amplify the *MAT2B* 3’-UTR. The PCR products were cloned into the pGL3 Luciferase Reporter Vector (Promega Italia, Milano, Italy) downstream from the firefly luciferase coding sequence. The *MAT2A* and *MAT2B* 3’-UTRs PCR constructs were checked using the ABI PRISM DNA sequencer. The mutagenesis of the target sequence of hsa-miR-203 in MAT2A and MAT2B 3’-UTRs was performed using the QuikChange site-directed mutagenesis kit (Agilent Technologies S.p.A., Milano, Italy) according to the manufacturer’s protocol. For the mutagenesis of MAT2A 3’-UTR, target site (+492-514) 5′-CCCUGUAACUUGAUCAACUCCUA-3′ was used. For the mutagenesis of the target site (+491-514) in the MAT2B 3’-UTR, 5′- UUUUUAUGCUUGAGAUAUUUCAA-3′ was used. All mutated sequences were validated and confirmed through DNA sequencing.

### Dual Luciferase Reporter assay

After transfection for 48 h cells were treated with lysing buffer and Luciferase assay was performed by Dual Luciferase Reporter Assay kit and the GloMax-Multi Detection System (Promega) according to the manufacturer’s protocol. The firefly luciferase activity was normalized relative to the Renilla luciferase activity.

### SAM, SAH and MTA determination

The high performance liquid chromatography analysis of SAM, SAH ad MTA content was made as reported [37]. For quantitative analysis of SAM, SAH and MTA content, the area of the chromatographic peaks in the tissue extracts was compared with that of standard solutions.

### Statistical analysis

Data are expressed as means ± SD. GraphPad Prism 5.01 (http://www.graphpad.com) was used to evaluate, by the Mann-Whitney, *U*-test, the significance of differences between means of qPCR and Western blot analyses of human samples, and calculate the correlation coefficient (R) by Spearman’s multiple regression analysis. Tukey-Kramer test was used to calculate the significance of differences between means of qPCR of rat samples. *P* < 0.05 was considered significant.

## SUPPLEMENTARY MATERIALS FIGURE


