# Supplementary material for: MicroRNA-203 impacts on the growth, aggressiveness and prognosis of hepatocellular carcinoma by targeting MAT2A and MAT2B genes
Source: Oncotarget. 2019 Apr 19;10(29):2835–54. doi: 10.18632/oncotarget.26838 (PMC6497462; doi:10.18632/oncotarget.26838)
Supplement: Supplementary file 1 [file oncotarget-10-2835-s001.pdf]

MicroRNA-203 impacts on the growth, aggressiveness and prognosis of hepatocellular carcinoma by targeting *MAT2A* and *MAT2B* genes

SUPPLEMENTARY MATERIALS

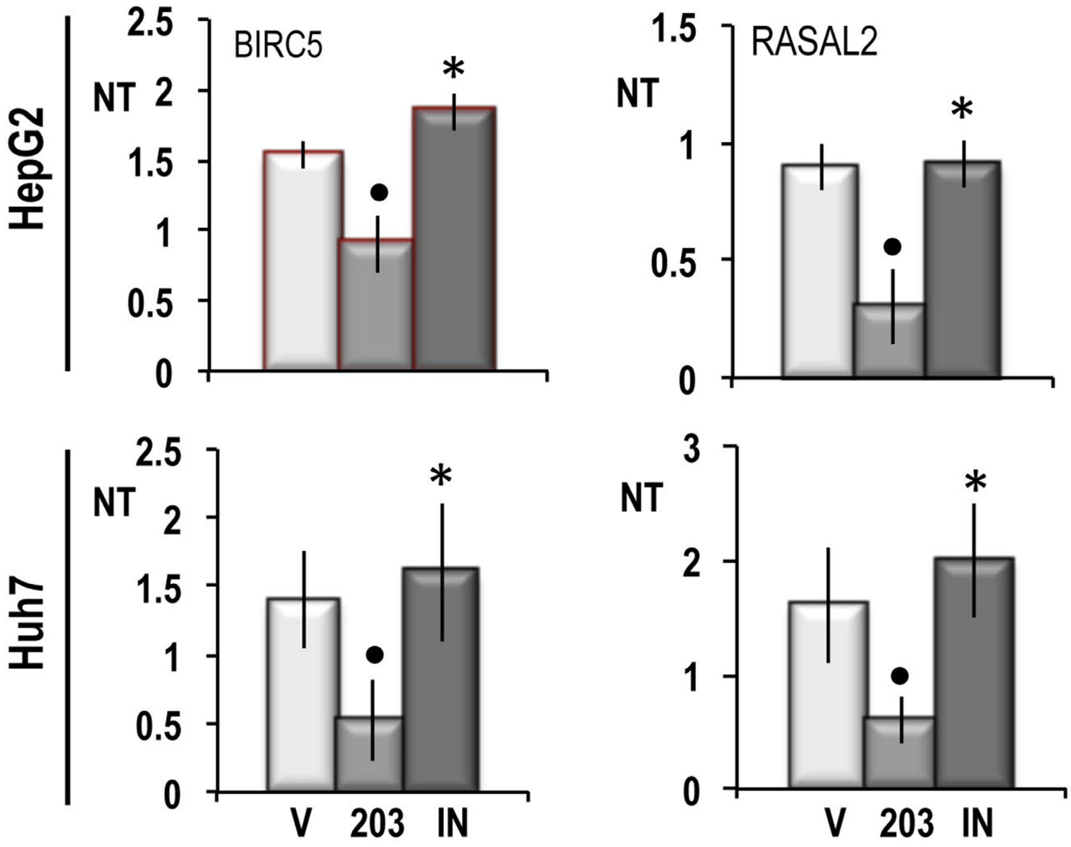

Supplementary Figure 1: Effects of the forced expression of miR-203 on BIRC5 and RASAL2 mRNA expression in HepG2 and Huh7 cells 48 h after transfection. The results are expressed as N-fold differences in target gene expression relative to the RNR-18 expression, named N Target (NT). NT = $2^{-\Delta CT}$, ΔCT of each sample was calculated by subtracting the Ct of the target gene from the Ct of the RNR-18 gene. Data are means ± standard deviation of 3 experiments. Mann-Whitney test: point, miR-203 vs. vector (V), at least $P < 0.05$; asterisk, miR-203 inhibitor vs. miR-203, $P < 0.001$.
